# The role of IL-31 and IL-34 in the diagnosis and treatment of chronic periodontitis

**DOI:** 10.1515/biol-2022-0563

**Published:** 2023-03-03

**Authors:** Ying Luo, Yi Ding, Yaduo Chen

**Affiliations:** Outpatient Department of Xiqu, West China Hospital of Stomatology, Sichuan University, Chengdu, China; Department of Periodontology, West China Hospital of Stomatology, Sichuan University, Chengdu, China

**Keywords:** chronic periodontitis, obesity, IL-31, IL-34, gingival crevicular fluid, serum

## Abstract

This study was devoted to demonstrating the role of IL-31 and IL-34 in the diagnosis and treatment of chronic periodontitis (CP). From the results, we found that the IL-31 and IL-34 levels were significantly increased in GCF and serum of CP patients compared with healthy controls or obese patients. Meanwhile, the area under the curve results further verified the diagnostic potential of IL-31 and IL-34 in terms of discriminating CP from obese patients at the GCF and serum levels. Finally, after 1 year of continuous treatment, we found that IL-31 and IL-34 levels were decreased in CP, suggesting their potential as biomarkers in CP treatment response. Monitoring GCF and serum levels of IL-31 and IL-34 contributed to CP detection and treatment response.

## 1 Introduction

Chronic periodontitis (CP) is a chronic disease whose pathogenesis is related to inflammation and the destruction of periodontal tissues due to the interaction between pathogenic bacteria and host immune responses. Studies have shown that many factors are associated with periodontitis, including living habits, eating habits, and obesity. Many previous studies have confirmed the relationship between obesity and periodontal disease; however, its mechanism has not been clearly identified. In 1997, according to the World Health Organization (WHO), scholars began to realize the harm of obesity [1]. Obesity is now thought to be a systemic chronic inflammatory disease caused by increased adipose tissues, whose etiology includes genetic factors, chronic infection, circadian rhythm changes, and so on [2].

In 2016, the American Society of Clinical Endocrinologists American Academy of Clinical Endocrinologists (AACE) published a gold standard for diagnosing obesity, including body mass index (BMI) and waist circumference [3]. In China, BMI ≥24 kg/m^2^ was defined as overweight, while BMI ≥28 kg/m^2^ was defined as obese [4]. Recently, nearly 604 million adults worldwide were obese, and at least 2.8 million people died each year from being overweight or obese [5]. As one of the most dangerous diseases threatening human health, obesity may cause many chronic diseases, including hypertension, hyperlipidemia, hyperglycemia, fatty liver, coronary heart disease, atherosclerosis, tumors, and periodontitis [6].

CP is a type of chronic infectious disease characterized by periodontal tissue destruction induced by plaque [7]. Currently, CP has become a major priority for healthcare systems around the world [8]. Data from the Centers for Disease Control and Prevention showed a 10–60% prevalence of CP in adults [9]. As a multifactor affecting disease, CP is mostly rooted in bacteria [10]. Hence, the basic therapy for CP is aimed at removing bacteria, including basic gingival cleaning and subgingival scaling [11]. At present, substantial evidence has demonstrated that obesity is a promoting factor of CP [12]. The general mechanism of CP caused by obesity is that obesity increases the release of inflammatory factors by upregulating oxidative stress products, thus promoting CP occurrence [12]. Miki et al. found that obesity can make periodontal tissue showing a more apparent inflammatory response [13]. However, the link between obesity and CP needs further study.

Interleukins (ILs) are a kind of cytokine produced by many cells and act on many cells [14,15]. IL plays an important role in transmitting information, activating and regulating immune cells, and mediating T- or B-cell activation, proliferation, and differentiation and in the inflammatory response. In obese patients, the expression of proinflammatory factors and anti-inflammatory factors is out of balance, so obese patients are in a low inflammatory state, which leads to the occurrence and development of CP. This study mainly illustrated the abnormal expression of IL-31 and IL-34 in CP and its clinical significance.

## 2 Methods

### 2.1 Inclusion and clinical data collection

This research was approved by and performed in accordance with the guidelines of the Medical Ethics Committee of Stomatology Hospital of Sichuan University (WCHSIRB-CT-2022-253). All subjects were enrolled from the Affiliated Hospital of Beihua University between December 2015 and April 2017. The clinical information of all subjects is shown in Table 1. Inclusion criteria were as follows: 18–60 years of age; adults capable of understanding and signing informed consent; and each patient had more than 15 teeth in the mouth. Study participation was agreed to by the subjects, and the informed consent form was signed. The diagnosis of CP was made with reference to the guidelines announced by the American Center for Disease Control and Prevention and the American Society of Periodontitis in 2007.

**Table 1:** Basic clinical information of all subjects

Parameters	Control group (*n* = 73)	Obese group (*n* = 69)	CP group (*n* = 138)
Age	53.205 ± 7.2	51.9 ± 6.8	52.4 ± 6.7
Gender (female/male)	36/37	33/36	72/66
Hypertension (%)	54.8	55.1	54.3
Smoking (%)	16.4	13.0	14.5
BMI (kg/m^2^)	23.6 ± 4.4	28.7 ± 3.6	29.8 ± 3.1*
Lost teeth number	0.5 ± 0.6	0.7 ± 0.4	2.2 ± 1.1*^,#^
PD (mm)	1.36 ± 0.51	1.42 ± 0.63	3.78 ± 0.71*^,#^
PLI	0.57 ± 0.38	0.51 ± 0.44	1.41 ± 0.53*^,#^
CAL (mm)	0.81 ± 0.42	0.93 ± 0.37	2.73 ± 0.29*^,#^
BOP (%)	9 ± 4	8 ± 3	38 ± 13*^,#^

Control: healthy subjects.

*: *P* < 0.01; *, #: *P* < 0.001.


**Informed consent:** Informed consent has been obtained from all individuals included in this study.
**Ethical approval:** The research related to human use has been complied with all the relevant national regulations, institutional policies, and in accordance with the tenets of the Helsinki Declaration and has been approved Medical Ethics Committee of Stomatology Hospital of Sichuan University (WCHSIRB-CT-2022-253).

### 2.2 Inclusion criteria

All the subjects were divided into three different groups: control group: probing depth (PD) <4 mm, plaque index (PLI) <1, clinical attachment loss (CAL) <1 mm, and BMI <24 kg/m^2^; obese group: PD <4 mm, PLI <1, clinical attachment loss (CAL) <1 mm, and BMI ≥28 kg/m^2^; and CP group: meet the diagnostic criteria of CP and BMI ≥28 kg/m^2^.

Exclusion criteria were as follows: pregnant and lactating women; patients with the orthodontic appliance; patients with periodontal treatment and lipid-lowering treatment in the last 6 months; patients with immunosuppressive drugs, nonhormonal anti-inflammatory drugs or antibiotics in the last 3 months; patients with heart disease; patients with hyperthyroidism or hypothyroidism and diabetes mellitus; and patients with mental illness.

### 2.3 Collection of gingival crevicular fluids and sera

For the collection of gingival crevicular fluids, the 1 and 2 quadrants of the first molar buccal tongue median were chosen as the 2 sites. After removing the large gingival stone of the tested tooth, the plaque on the side was scraped with the tip probe, and the tip of the 25th hygroscopic paper was gently inserted into the bottom of the periodontal bag, staying for 1 min. The tip of the paper was placed in the EP tube, and then, 300 μL of PBS buffer was added and stored at −80℃.

For collection of sera, 2 mL of peripheral blood was collected, fully coagulated, and centrifuged at 2,000 rpm for 20 min. Then, the supernatants were collected and stored at −80°C until use.

### 2.4 Evaluation of periodontal clinical parameters

The basic clinical information of all subjects, including age, sex, hypertension, smoking, BMI, lost teeth, PD, PLI, clinical attachment level (CAL), and bleeding on probing (BOP), was collected and analyzed.

### 2.5 Evaluation of IL-31 and IL-34 levels measured by ELISA

All samples were stored at room temperature for 20 min and centrifuged at 4°C for 10 min. The supernatant was taken for examination after centrifugation, and the levels of IL-31 and IL-34 in GCF and serum were detected by corresponding ELISA kits (Jiangsu Meimian Industrial Co., Ltd, Jiangsu, China). The absorbance values were determined using a microplate reader at a wavelength of 450 nm, and the concentration was calculated according to the standard curve.

### 2.6 Receiving operating characteristics (ROC) analysis

The area under the curve (AUC) of the ROC analysis was between 0.5 and 1.0. The closer the AUC was to 1, the better the diagnostic effect was. High accuracy was defined as an AUC of more than 0.9. However, an AUC result of less than 0.9 was considered to have low accuracy.

### 2.7 Statistical analysis

SPSS 20.0 software (SPSS Inc., Chicago, IL, USA) and GraphPad Prism 6.0 software were used for statistical analysis in this study. One-way analysis of variance followed by Tukey’s *post hoc* test was carried out to compare data among the healthy control, normal CP and CP groups. The diagnostic impacts of IL-31 and IL-34 were verified by ROC analysis. A *P* value of less than 0.05 was deemed statistically significant.

## 3 Results

### 3.1 Clinical parameters of all subjects

**Figure 1:**
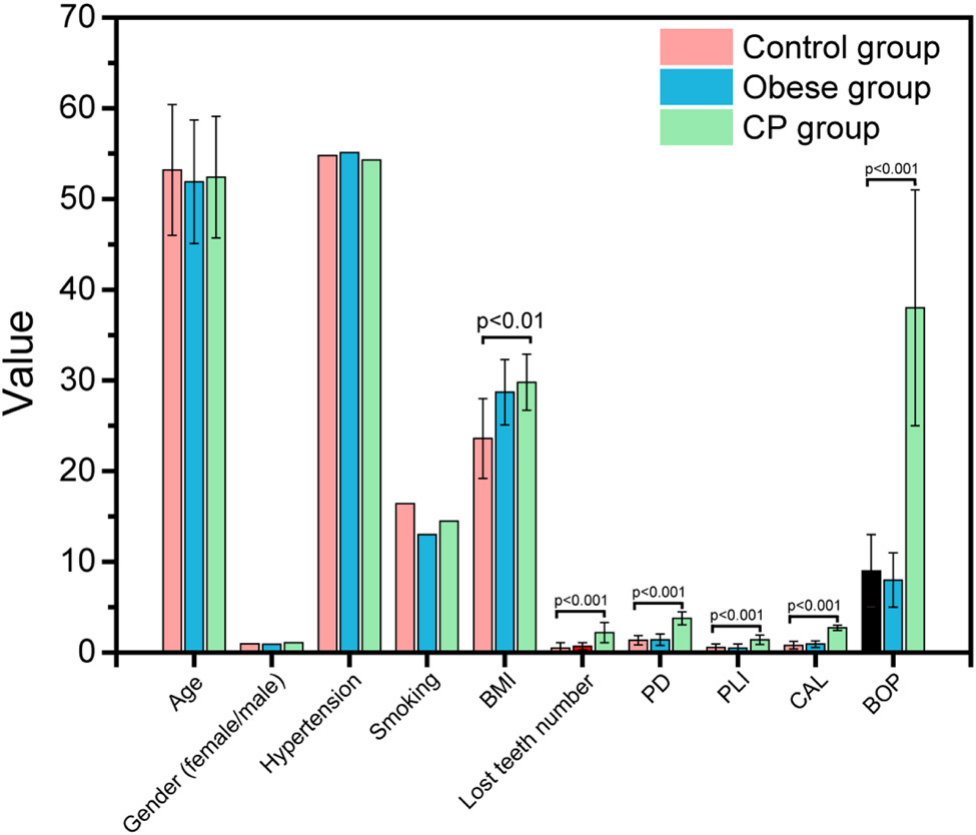
Parameters of the control group, obese group and CP group.

**Figure 2:**
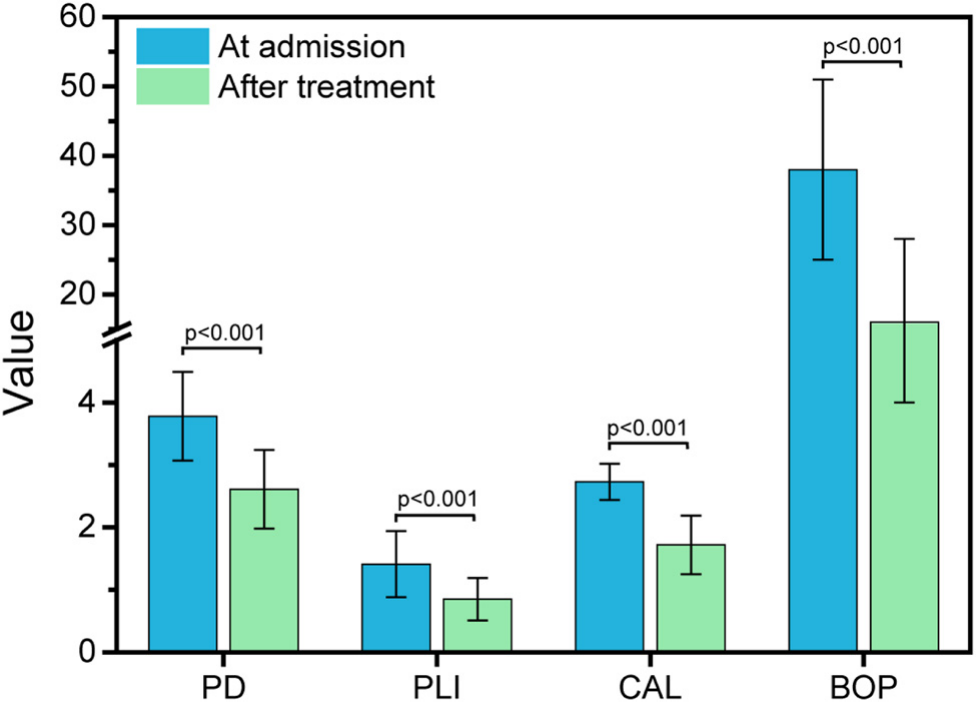
PD, PL1, CAL, and BOP of periodontal clinical parameters in CP at admission and after treatment.

The basic clinical information among all subjects was recorded and analyzed. As shown in Table 1 and Figure 1, there were significant differences in BMI, lost teeth number, PD, PLI, CAL, and BOP between the CP and control groups; however, no significant differences were found in age, sex, hypertension, or smoking. Furthermore, after 1 year of treatment, the periodontal clinical parameters in CP were recorded and compared with pretreatment conditions. As shown in Table 2 and Figure 2, the PD, PLI, CAL, and BOP in CP patients after treatment were significantly alleviated, suggesting that the treatment was effective.

**Table 2:** Comparison of periodontal clinical parameters in CP at admission and after treatment

Parameters	At admission	After treatment	*t* value	*P* value
PD (mm)	3.78 ± 0.71	2.61 ± 0.63	14.4798	<0.0001
PLI	1.41 ± 0.53	0.85 ± 0.34	10.4473	<0.0001
CAL (mm)	2.73 ± 0.29	1.72 ± 0.47	21.4838	<0.0001
BOP (%)	38 ± 1,338	16 ± 12	14.6080	<0.0001

### 3.2 Eccentric levels of IL-31 and IL-34 in GCF and serums of subjects

**Figure 3:**
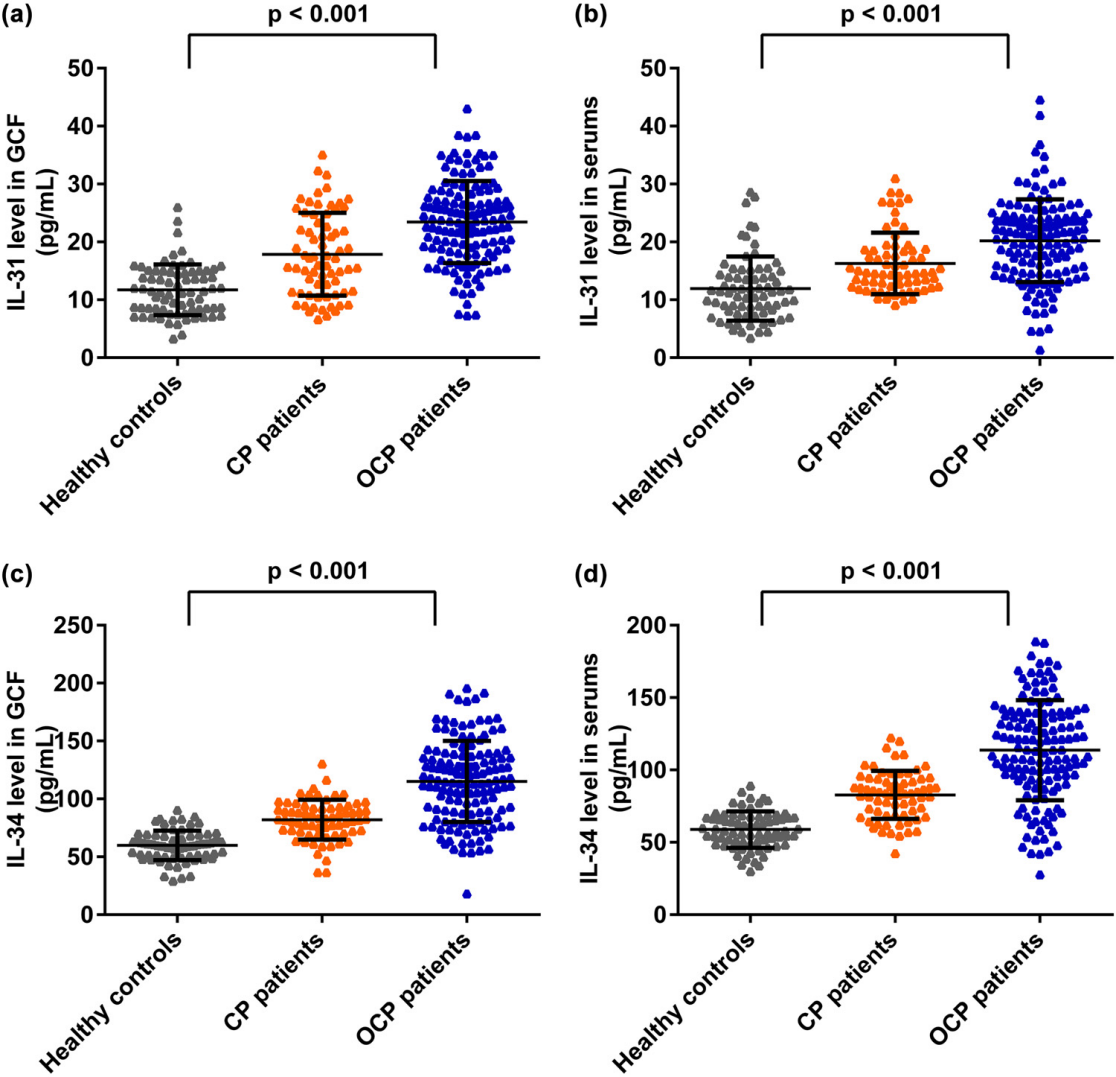
IL-31 and IL-34 levels in GCF and serum of subjects. (a) IL-31 levels in GCF. (b) IL-31 levels in serum. (c) IL-34 levels in GCF. (d) IL-34 levels in serum. GCF: gingival crevicular fluids; CP: chronic periodontitis.

The expression levels of IL-31 and IL-34 in GCF and serum samples obtained from all subjects are compared in Figure 3. As shown, the expression levels of IL-31 and IL-34 in GCF and serum were both increased in the obese and CP groups. Meanwhile, IL-31 and IL-34 were significantly increased in the CP group in both GCF and serum levels (***P < 0.001). However, there was no significant difference between the obese and control groups.

### 3.3 ROC results of IL-31 and IL-34 in CP

**Figure 4:**
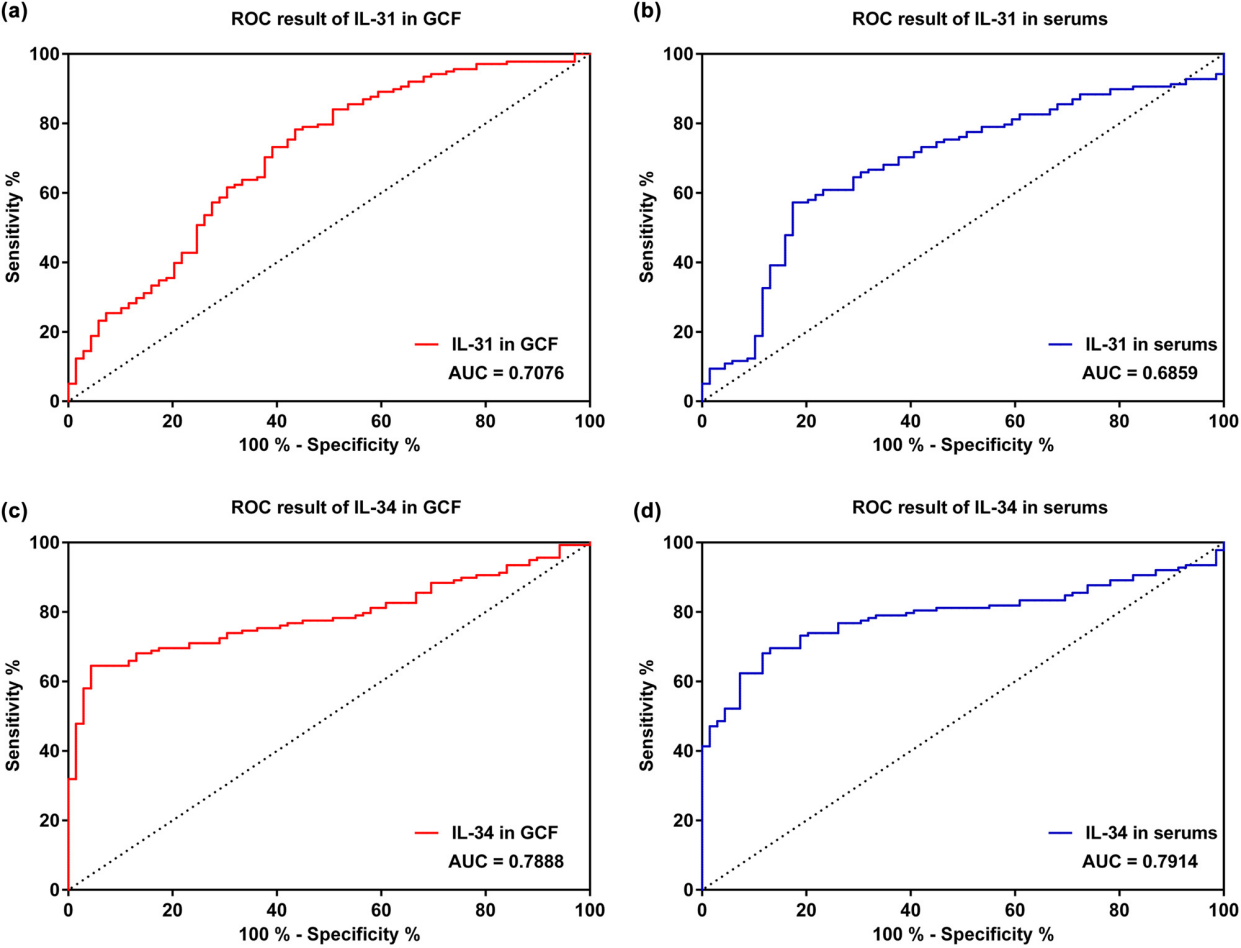
ROC results of IL-31 and IL-34 in distinguishing CP patients from obese patients. (a) IL-31 in GCF discriminating CP patients from obese patients. AUC = 0.7076, 95% CI = 0.6309–0.7844. (b) Serum levels of IL-31 discriminating CP patients from obese patients. AUC = 0.6859, 95% CI = 0.6091–0.7627. (c) IL-34 in GCF discriminating CP patients from obese patients. AUC = 0.7888, 95% CI = 0.7286–0.8490. (d) Serum levels of IL-34 discriminating CP patients from obese patients. AUC = 0.7914, 95% CI = 0.7309–0.8520. GCF: gingival crevicular fluids; CP: chronic periodontitis.

The AUCs of IL-31 and IL-34 in CP were determined by ROC analysis. As shown in Figure 4, the ROC results of IL-31 and IL-34 in GCF were 0.7076 (95% CI = 0.6309–0.7844) and 0.7888 (95% CI = 0.7286–0.8490), respectively. Meanwhile, the AUCs of IL-31 and IL-34 in serum were 0.6859 (95% CI = 0.6091–0.7627) and 0.7914 (95% CI = 0.7309–0.8520), respectively.

### 3.4 Changes in IL-31 and IL-33 levels before and after treatment in CP patients

**Figure 5:**
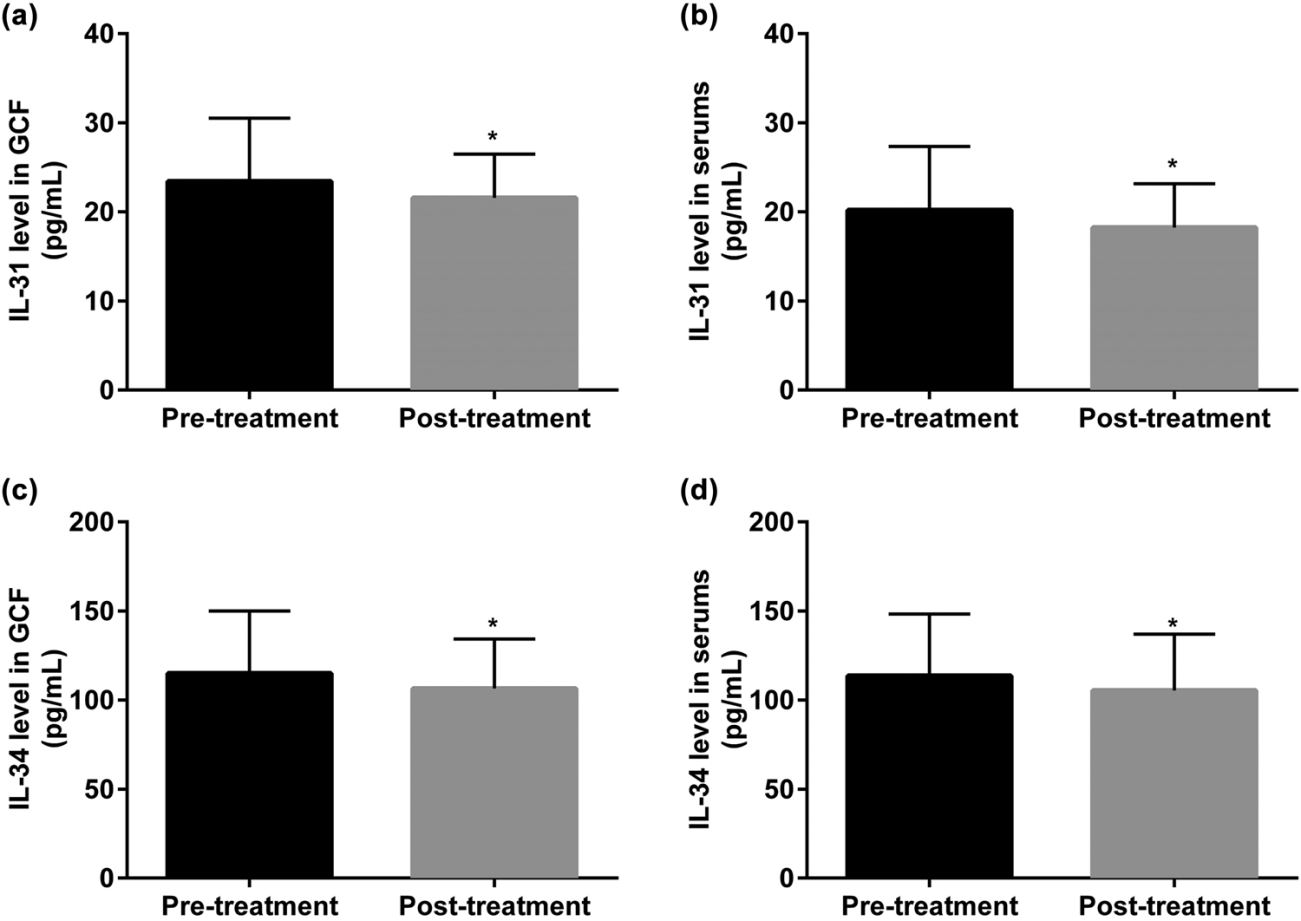
The variation tendency of IL-31 and IL-34 in CP at admission and after treatment. (a) Changes in IL-31 in GCF before and after treatment in CP. (b) Changes in IL-31 in serum before and after treatment in CP. (c) Changes in IL-34 in GCF before and after treatment in CP. (d) Changes in IL-34 in serum before and after treatment in CP. GCF: gingival crevicular fluids; CP: chronic periodontitis.

To confirm the differences in IL-31 and IL-34 levels before and after treatment in CP patients, we detected GCF and serum levels from CP patients at admission and 1 year after treatment. From the results in Figure 5, the expressions of IL-31 and IL-34 were decreased after a 1-year treatment, compared with the pretreatment group (*P < 0.05).

## 4 Discussion

CP is one of the leading causes of tooth loss among adults and endangers human teeth and general health [16]. The chronic inflammation of periodontitis causes not only tooth loss but also the absorption of alveolar bone, which makes the repair of missing teeth more difficult [17]. Due to its slow development and lack of obvious symptoms, many CP patients may be diagnosed several years after occurrence [18]. Its long-term chronic inflammatory effects produce many pathological changes in the oral cavity and multiorgan systems of the whole body [19]. To date, systemic diseases reported to be affected or induced by CP include diabetes, osteoporosis, and arthritis [20]. Hence, the mechanisms of CP diagnosis and treatment have become a hot spot in clinical research.

In recent years, studies have shown that obesity is closely associated with CP and that overweight or obese people have a higher risk of CP [21]. Obesity is often accompanied by changes in the microbial community in the body; meanwhile, changes in intestinal microbes directly affect appetite, intestinal absorption, and immune function [22]. The systematic evaluation and meta-analysis published by Chaffee and Weston identified a positive correlation between obesity and CP; excluding other interference factors with periodontitis, the incidence of CP patients with obesity was 2–3 times that of the average population [23]. Another study showed that the number of remaining teeth and the periodontal bag depth were significantly correlated with BMI, fat content, and waist circumference [24]. A cross-sectional study in Brazil showed that obese patients had a higher risk of CP [12]. A 5-year follow-up study of 3,590 individuals in Japan also found a linear correlation between CP progression and BMI [25]. A 40-year study of 1,038 veterans in the USA found that BMI, waist circumference, and waist circumference–height ratio can be used as promising predictors of CP progression; meanwhile, for people who gained more weight, the degree of periodontal attachment loss was more serious. In obese people, the expression of proinflammatory and anti-inflammatory factors is out of balance, thus forming a chronic inflammatory state. A study clarified that after weight loss, the levels of inflammatory factors in GCF in CP patients were decreased significantly, suggesting a potential relationship between inflammatory cytokines and CP development [26].

IL-34 is a new cytokine identified in embryonic kidney cell lines by Greter et al. in 2008 [27]. IL-31, a newly discovered cytokine in 2004, is a single-stranded molecule with four helical structures that is mainly produced by activated T cells [28]. The expression of IL-31 and IL-34 in the gingival tissue of patients with severe periodontitis was significantly higher than that of patients with mild periodontitis, indicating that IL-31 and IL-34 may be closely related to the severity of periodontitis. Macrophages can secrete IL-6, TNF-α, and other cytokines under pathological conditions, which can regulate immune and inflammatory responses [29]. As a functional ligand of macrophage colony-stimulating factor receptor (M-CSFR), IL-34 can combine with M-CSFR to activate downstream signaling pathways and exert biological functions [30]. Research has shown that TNF-α can stimulate osteoblasts to express IL-34 and replace human macrophage colony-stimulating factor (M-CSF) to bind to human M-CSFR to activate the extracellular signal pathway. In response to RANKL, it could promote the formation of osteoclasts and lead to bone absorption. IL-34 could promote monocytes to develop toward macrophages. After periodontitis, IL-34 promotes monocyte development toward macrophages, and macrophages secrete a series of cytokines, such as IL-6 and TNF-α, mediating the inflammatory response. TNF-α secreted by macrophages further stimulates osteoblasts to express IL-34. Finally, IL-34 can promote the formation of osteoclasts by combining with M-CSFR, leading to bone resorption and thus accelerating the development of periodontitis. A variety of reports have revealed the association between IL-31, IL-33, and inflammation processes. For instance, Rosanna et al. described the potential roles of IL-31 and IL-34 in immune responses [31]. Moreover, Hou et al. reported that IL-31 may induce proinflammatory cytokines and participate in proinflammatory processes in many diseases [32]. Miake et al. demonstrated that IL-31 could elevate the expression of several inflammatory chemokines and play an essential role in the lung inflammation process [33]. Another study illustrated that IL-34 was elevated in gestational diabetes mellitus and may cause inflammation by targeting GSF-1R [34]. However, the roles of IL-31 and IL-34 in CP remain unknown. In the present study, we found that IL-31 and IL-34 were prominently increased in CP patients compared with obese or healthy participants; moreover, ROC analysis further verified the potential of IL-31 and IL-34 in the diagnosis of CP as well. In addition, after 1 year of treatment, the expression levels of IL-31 and IL-34 were decreased in CP patients along with alleviated PD, PLI, CAL, and BOP indexes compared with pretreatment conditions.

## 5 Conclusion

IL-31 and IL-34 play essential roles in the detection and treatment response of CP, expanding the landscape for CP clinical treatment. A limitation of this study is the link between periodontitis and systemic diseases.
